# Decremental conduction property in the slow conduction zone of adenosine‐sensitive atrial tachycardia

**DOI:** 10.1002/joa3.13140

**Published:** 2024-08-27

**Authors:** Takahiko Kinjo, Masaomi Kimura, Noriyoshi Kaname, Daisuke Horiuchi, Hirofumi Tomita

**Affiliations:** ^1^ Department of Cardiology and Nephrology Hirosaki University Graduate School of Medicine Hirosaki Japan; ^2^ Present address: Department of Pediatric Cardiology and Adult Congenital Cardiology Tokyo Women's Medical University Tokyo Japan

**Keywords:** adenosine‐sensitive atrial tachycardia, atrioventricular node‐like tissue, decremental conduction, entrainment

## Abstract

In the case of adenosine‐sensitive atrial tachycardia originating near the atrioventricular (AV) node, overdrive pacing from the anterior right atrium showed constant and progressive fusion, indicating that the pacing site is proximal to slow conduction. Shortening the pacing cycle length prolonged conduction times to the orthodromic capture sites; they remained unchanged at the antidromic capture sites. Limited decremental conduction property in the slow conduction zone supports the hypothesis that AV node‐like tissue remnants along the AV annulus are involved.

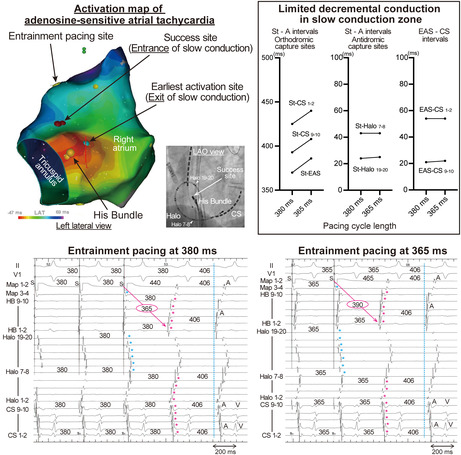

Adenosine‐ (or verapamil‐) sensitive atrial tachycardia (AT), which originates near the atrioventricular (AV) node, has been reported as a reentrant AT involving calcium channel‐dependent tissues.^1^, ^2^ Previous studies have shown that manifest entrainment with fusion (P wave and/or intracardiac electrograms) of adenosine‐ (or verapamil‐) sensitive AT was demonstrable and successfully ablated at the site, which is thought to be the entrance to the slow conduction zone.^3^, ^4^ Here, we describe a case of adenosine‐sensitive AT, which showed decremental conduction properties in the slow conduction zone.

An 84‐year‐old man without structural heart disease or electrocardiogram abnormalities (Figure 1A) suffered from palpitation and presyncope. Electrocardiography revealed supraventricular tachycardia (Figure 1B). He was admitted to our hospital for catheter ablation after failed medical therapy. Ventriculoatrial dissociation was observed in ventricular overdrive pacing during tachycardia and during sinus rhythm, suggesting that AV nodal reentrant tachycardia or AV‐reciprocating tachycardia were unlikely. An intravenous 5 mg bolus dose of adenosine 5′‐triphosphate terminated the tachycardia without AV block. The electroanatomical activation map of the right atrium showed a centrifugal activation pattern with the earliest activation site (EAS) of tachycardia observed near the His bundle (HB) (Figure 1C). After a duodecapolar electrode catheter (Halo) positioned at the tricuspid annulus, overdrive pacing during the tachycardia from the anterior right atrium showed manifest entrainment (Figure 1D). The HB catheter was located at the EAS and was orthodromically captured by a long stimulus to the (St‐) EAS interval during entrainment. Immediately thereafter, overdrive pacing from the same site at a slightly shorter cycle length resulted in a progressive fusion of the P wave with a prolonged St‐EAS interval (Figure 1E). Conduction times over the St‐atrium were prolonged in the orthodromic capture sites (EAS and CS), while they were unchanged in the antidromic capture sites (Halo 7–8, Halo 19–20) (Figure 2 left and middle panel). Intervals of EAS to CS were also unchanged (Figure 2 right panel). Constant and progressive fusion of the P wave during overdrive atrial pacing indicates that the mechanism of the AT is reentry. Tachycardia was diagnosed as adenosine‐sensitive AT, in which the entrainment pacing site and EAS were assumed to be the proximity and exit of the slow conduction zone, respectively. Radiofrequency ablation between the entrainment pacing site and the EAS successfully terminated the AT (Figure 3A,B). After ablation, the AT was no longer inducible, and the patient remained free from AT for 2 years.

**FIGURE 1:**
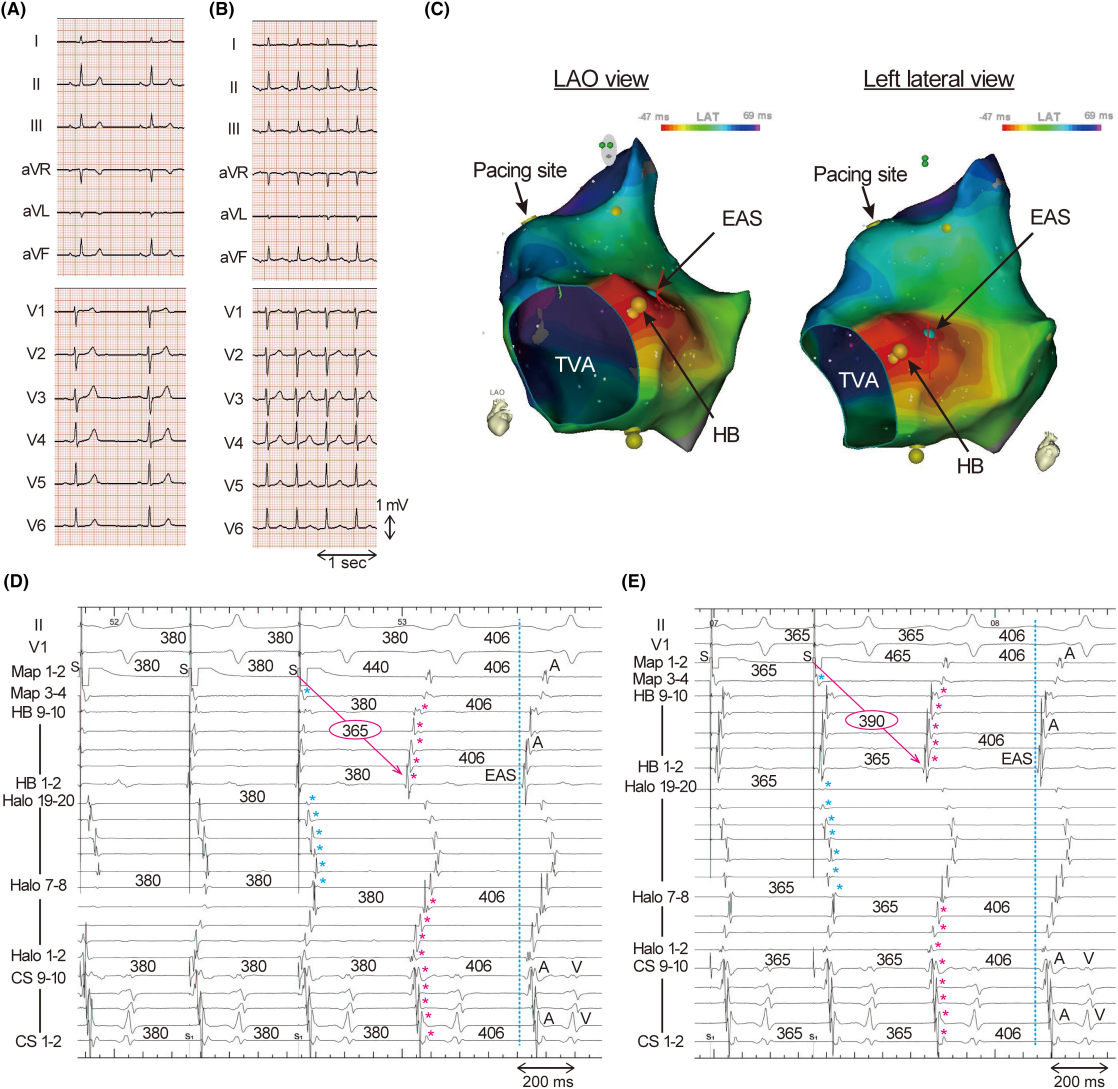
Electrocardiogram during sinus rhythm (A) and tachycardia (B). (C) Electroanatomical activation map of the tachycardia. (D) Atrial overdrive pacing during tachycardia. The asterisks indicate the last atrial electrograms captured by the last pacing stimulus. Constant fusion is clarified by intracardiac electrograms. Note the His bundle (HB) catheter is pulled back into the right atrium to record the electrogram of the earliest activation site (EAS) (cyan dotted line). (E) Atrial overdrive pacing during tachycardia at shorter cycle length. Note the P wave during entrainment differs from Figure 1D, indicating progressive fusion. CS, coronary sinus; EAS, earliest activation site; HB, His bundle; LAO, left anterior oblique; TVA, tricuspid valve annulus.

**FIGURE 2:**
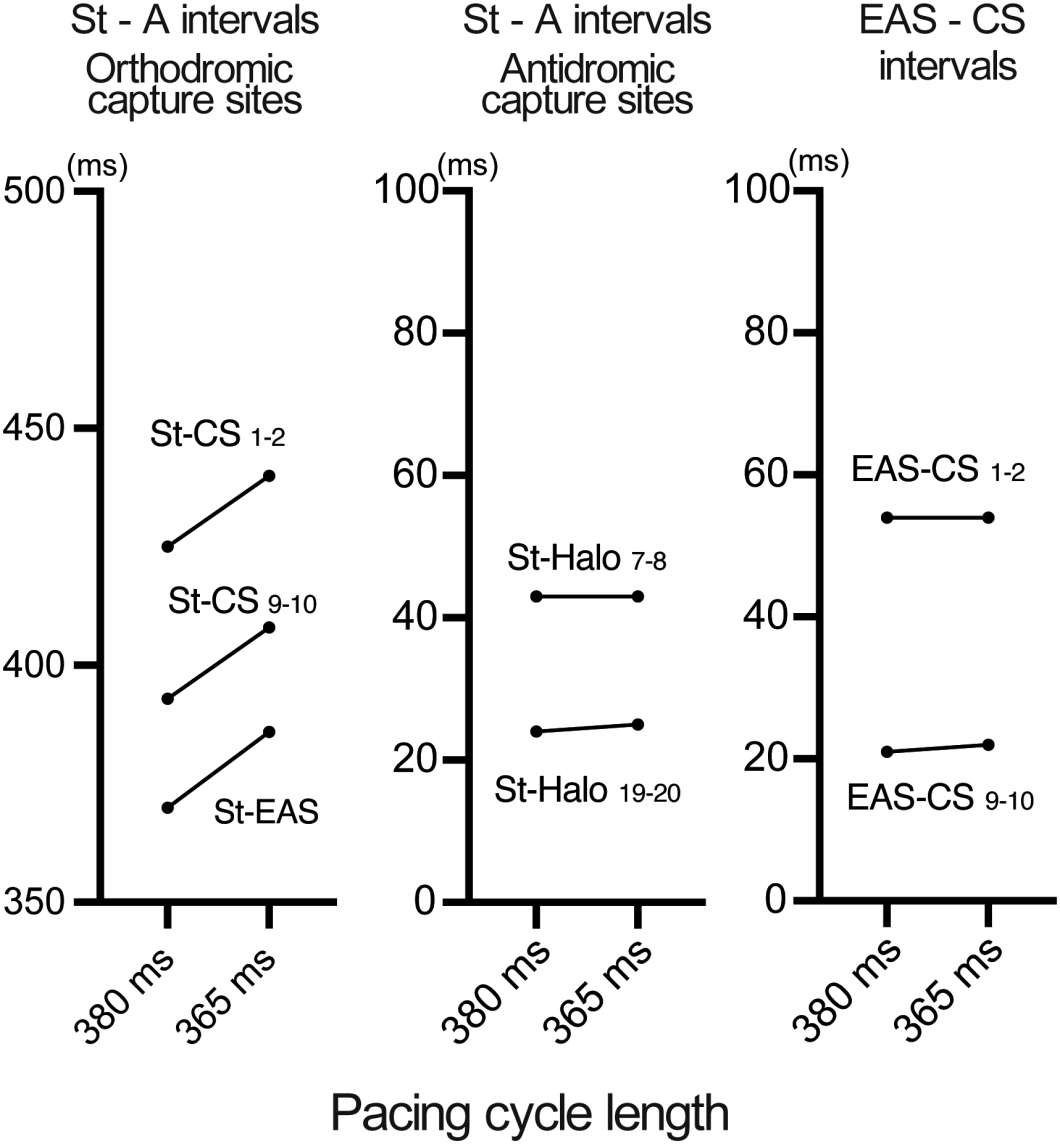
Intervals of stimulus to atrium (St‐A) of orthodromic capture sites (left panel), antidromic capture sites (middle panel), and the EAS to CS (right panel) during entrainment. All abbreviations are as in Figure 1.

**FIGURE 3:**
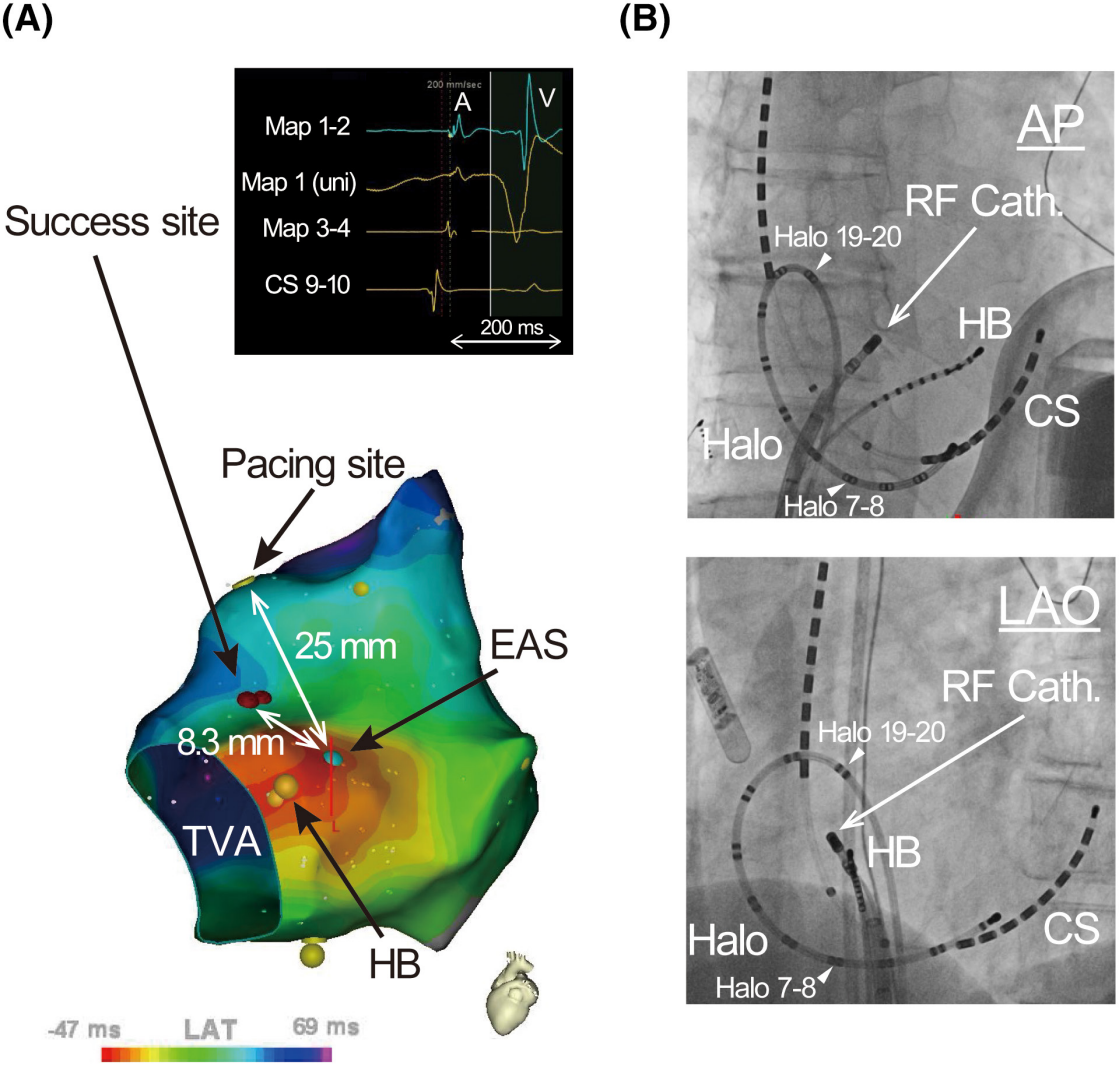
(A) Demonstration of successful ablation site. Note that electrograms of atrium and ventricle are recorded, indicating this site is atrioventricular annulus. (B) Fluoroscopy images show the location of the catheters. AP, anterior–posterior; St‐, stimulus to; other abbreviations are as in Figure 1.

This case demonstrates that the slow conduction zone of adenosine‐sensitive AT shows a decremental conduction property, which supports the hypothesis that remnants of the AV node‐like tissues along the AV annulus are involved.^4^ In a previous report, postpacing interval (PPI) was equal to the tachycardia cycle length (TCL) when overdrive pacing (increasing by 5 beats/min) from the EAS, while PPI was prolonged compared with the TCL when overdrive pacing at increasing by 10 beats/min.^2^ Therefore, decremental conduction may occur somewhere in the tachycardia circuit. Furthermore, our case demonstrated that the decremental conduction property was limited in the slow conduction zone; delay in the atrial myocardium or local pacing latency was ruled out because the St‐atrium intervals of antidromic capture sites and intervals of EAS to CS (1–2 and 7–8), both of which are considered outside the slow conduction zone, were unchanged after shortening the pacing cycle length. In addition, electrograms of the atrium and ventricle were recorded at the success site (Figure 3), indicating that the entrance of the slow conduction zone is located in the AV annulus.

Our case revisited the electrophysiological features of adenosine‐sensitive AT and pointed out the potential pitfalls. For example, a PPI equal to the TCL is a widely accepted criterion indicating that the pacing site is on the circuit. However, the decremental conduction property can misleadingly prolong the PPI, even when the pacing site is on the tachycardia circuit.^5^

In conclusion, decremental conduction was demonstrated in the slow conduction zone of adenosine‐sensitive AT, which supports the hypothesis that remnants of AV node‐like tissue along the AV annulus are involved.

## FUNDING INFORMATION

This report received no specific grant from public, commercial, or not‐for‐profit funding agencies.

## CONFLICT OF INTEREST STATEMENT

Dr. Masaomi Kimura is an associate professor of the Department of Advanced Management of Cardiac Arrhythmias, which is an endowment department supported by Medtronic Japan Co., Ltd., and Fukuda Denshi Kita‐Tohoku Hanbai Co., Ltd. Dr. Hirofumi Tomita is a concurrent professor of the Department of Advanced Management of Cardiac Arrhythmias and also received a research grant from Abbott Medical Japan LLC. Other authors have no relevant disclosures.

## ETHICS APPROVAL STATEMENT

None.

## PATIENT CONSENT STATEMENT

The patient gave informed consent to publish this case report.

## PERMISSION TO REPRODUCE MATERIAL FROM OTHER SOURCES

None.

## CLINICAL TRIAL REGISTRATION

None.

## Data Availability

Data sharing is not applicable to this article because no datasets were generated or analyzed during this report.

## References

[1] Iesaka Y , Takahashi A , Goya M , Soejima Y , Okamoto Y , Fujiwara H , et al. Adenosine‐sensitive atrial reentrant tachycardia originating from the atrioventricular nodal transitional area. J Cardiovasc Electrophysiol. 1997;8:854–864. 10.1111/j.1540-8167.1997.tb00846.x 9261711

[2] Yamabe H , Tanaka Y , Okumura K , Morikami Y , Kimura Y , Hokamura Y , et al. Electrophysiologic characteristics of verapamil‐sensitive atrial tachycardia originating from the atrioventricular annulus. Am J Cardiol. 2005;95:1425–1430. 10.1016/j.amjcard.2005.02.007 15950564

[3] Yamabe H , Okumura K , Morihisa K , Koyama J , Kanazawa H , Hoshiyama T , et al. Demonstration of anatomical reentrant tachycardia circuit in verapamil‐sensitive atrial tachycardia originating from the vicinity of the atrioventricular node. Heart Rhythm. 2012;9:1475–1483. 10.1016/j.hrthm.2012.05.012 22583842

[4] Okumura K , Sasaki S , Kimura M , Horiuchi D , Sasaki K , Itoh T , et al. Usefulness of combined CARTO electroanatomical mapping and manifest entrainment in ablating adenosine triphosphate‐sensitive atrial tachycardia originating from the atrioventricular node vicinity. J Arrhythm. 2016;32:133–140. 10.1016/j.joa.2015.11.004 27092195 PMC4823578

[5] Kinjo T , Sasaki S , Kimura M , Owada S , Horiuchi D , Sasaki K , et al. Long postpacing interval after entrainment of tachycardia including a slow conduction zone within the circuit. J Cardiovasc Electrophysiol. 2016;27:923–929. 10.1111/jce.13014 27196507

